# Synthesis of a Pd_2_L_4_ Hydrazone Molecular Cage Through Multiple Reaction Pathways

**DOI:** 10.3390/ijms252211861

**Published:** 2024-11-05

**Authors:** Giovanni Montà-González, Ramón Martínez-Máñez, Vicente Martí-Centelles

**Affiliations:** 1 Instituto Interuniversitario de Investigación de Reconocimiento Molecular y Desarrollo Tecnológico (IDM), Universitat Politècnica de València, Universitat de València, Camino de Vera s/n, 46022 Valencia, Spain; gmongon@upv.es; 2 Departamento de Química, Universitat Politècnica de València, Camino de Vera s/n, 46022 Valencia, Spain; 3 CIBER de Bioingeniería, Biomateriales y Nanomedicina (CIBER-BBN), Instituto de Salud Carlos III, 28029 Madrid, Spain; 4 Unidad Mixta de Investigación en Nanomedicina y Sensores, Universitat Politècnica de València, Instituto de Investigación Sanitaria La Fe (IISLAFE), Avenida Fernando Abril Martorell 106, 46026 Valencia, Spain; 5 Unidad Mixta UPV-CIPF de Investigación en Mecanismos de Enfermedades y Nanomedicina, Universitat Politècnica de València, Centro de Investigación Príncipe Felipe, Avenida Eduardo Primo Yúfera 3, 46012 Valencia, Spain

**Keywords:** molecular cages, supramolecular chemistry, metal-organic cages, cage synthesis

## Abstract

Molecular cages are preorganized molecules with a central cavity, typically formed through the reaction of their building blocks through chemical bonds. This requires, in most cases, forming and breaking reversible bonds during the cage formation reaction pathway for error correction to drive the reaction to the cage product. In this work, we focus on both Pd–ligand and hydrazone bonds implemented in the structure of a Pd_2_L_4_ hydrazone molecular cage. As the cage contains two different types of reversible bonds, we envisaged a cage formation comparative study by performing the synthesis of the cage through three different reaction pathways involving the formation of Pd–ligand bonds, hydrazone bonds, or a combination of both. The three reaction pathways produce the cage with yields ranging from 73% to 79%. Despite the complexity of the reaction, the cage is formed in a high yield, even for the reaction pathway that involves the formation of 16 bonds. This research paves the way for more sophisticated cage designs through complex reaction pathways.

## 1. Introduction

Molecular cages are preorganized hosts with a central cavity that provides enhanced host–guest properties compared to less preorganized systems such as macrocycles [1,2], aiming to mimic the sophisticated cavity and functions of enzymes [3,4,5,6]. Chemists have developed synthetic methods to prepare both metal-organic cages and purely organic cages, resulting in a wide range of structures with size- and shape-dependent host–guest properties [7,8,9]. Encapsulation in the cavity of cages results in different effects on the guest molecule, ranging from activation for catalytic reactions to protection from the surrounding media. These effects yielded multiple applications of molecular cages, including catalysis [10,11,12,13,14], sensing of chemicals [15,16,17,18,19,20], stabilization of chemical species [21,22], separation process [23,24,25], removal of pollutants from water [26,27,28,29] and biological applications [30,31,32,33,34,35,36,37,38,39,40] among many others [7,8,41,42].

The synthesis of molecular cages from the constituent building block involves in most cases numerous reversible steps. Reversibility is key for error correction of improperly formed by-products during the cage formation process [43,44,45]. For this, the cage building blocks must have a specific shape and geometry that provides an appropriate preorganization in a similar fashion to macrocycles [7,46]. Besides the geometric requirements of the building blocks, both thermodynamic and kinetic aspects are key to setting up the appropriate reaction conditions, including concentration of reagents, reaction time, and temperature [43,44,45,47]. In this regard, computational modeling has been extensively used to design cages with specific geometries and properties such as predicting host-guest affinity [48,49,50,51,52,53,54].

Typically, metal-organic cages are prepared by the reaction of ligands with metals forming metal–ligand bonds [8,55,56], and purely organic cages are prepared through the reaction of ligands with complementary reactivity through reversible reactions such as imine and hydrazone bond formation [7,57,58]. There are examples of cages containing both metal–ligand bonds and reversible organic bonds, allowing their formation through either type of bond, or even both simultaneously [8,59,60,61] Focusing in Pd(II) containing cages, the formation of Pd_2_L_4_ cages involves the reaction of two Pd(II) ions and four ditopic ligands, typically containing pyridine moieties [62,63,64,65], though Pd–pyridine coordination bonds [66]. Regarding hydrazone-containing cages, hydrazone bonds are both reversible and robust [67], allowing the preparation of cages through the condensation reaction between hydrazide and aldehyde-containing building blocks [31,68,69,70,71,72,73,74,75,76]. Combining both strategies, Crowley and his team showed the feasibility of simultaneously using the cage synthesis of both Pd–pyridine coordination bonds and hydrazone bonds [60]. Similarly, we proved that it is possible to synthesize cages that contain Pd–pyridine bonds through hydrazone bond formation [31]. These results open the way to explore multiple cage formation pathways involving both Pd–pyridine and hydrazone bonds. For this, we propose studying the synthesis of a Pd_2_L_4_ cage through different reaction pathways involving the formation of Pd–ligand bonds, hydrazone bonds, or a combination of both.

**Figure 1:**
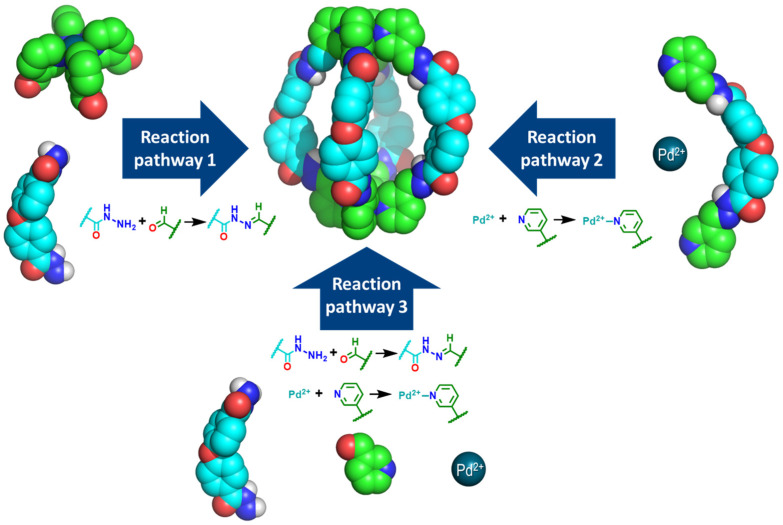
Schematic representation of the three reaction pathways for the synthesis of a Pd_2_L_4_ cage containing hydrazone and Pd–pyridine bonds. In the figura, carbon atoms in nicotinaldehyde are shown in green, while carbon atoms in 4,4′-oxydi(benzohydrazide) are colored cyan. Oxygen atoms are represented in red, nitrogen atoms in blue, hydrogen atoms in white, and Pd^2+^ ions in dark cyan.

In this work, we present a comparative study of the synthesis of a Pd_2_L_4_ cage containing Pd–pyridine and hydrazone bonds [31]. The reversible nature of both bond types enables the formation of the cage through the formation of Pd–ligand bonds or hydrazone bonds, utilizing three distinct reaction pathways (Figure 1). As far as we know, this is the first cage formation comparative study involving Pd–ligand and organic bond formation reactions. In particular, we focused on a Pd_2_L_4_ cage containing four dihydrazone units with a bent geometry and two [PdPy_4_]^2+^ units with a *C*_4_ symmetric geometry that serve to cap the cage [31].

## 2. Results and Discussion

### 2.1. Synthesis of the Pd_2_L_4_ Cage

**Figure 2:**
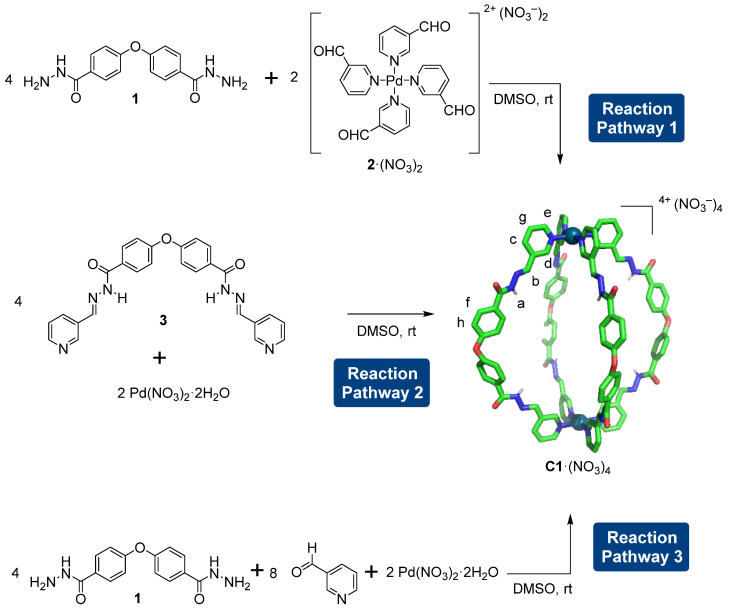
The three possible reaction pathways for the synthesis of cage **C1**·(NO_3_)_4_ by Pd–pyridine and hydrazone bond formation. The lettering of **C1**·(NO_3_)_4_ corresponds to the assignment of the ^1^H NMR signals. The molecular model of **C1**·(NO_3_)_4_, in which non-polar hydrogen atoms have been omitted for clarity, has the following color scheme: C, green; O, red; N, blue; H, white; and Pd^2+^, dark cyan. Note that **C1** = [Pd_2_L_4_]^4+^; therefore, four nitrate counterions are required, i.e., **C1**·(NO_3_)_4_.

The synthesis of the Pd_2_L_4_ cage that contains both Pd–pyridine and hydrazone bonds can be performed in multiple ways. In this regard, we proposed three possible reactions to prepare the Pd_2_L_4_ cage **C1**·(NO_3_)_4_ (Figure 2). The reaction pathway 1 involves the reaction of dihydrazide **1** and the square planar tetranicotinaldehyde palladium(II) nitrate motif **2** by hydrazone bond formation [31]; the reaction pathway 2 comprises the reaction of dipyridine ligand **3** with palladium(II) nitrate dihydrate by Pd–pyridine bond formation; and the reaction pathway 3 involves the reaction between dihydrazide 4,4′-oxydi(benzohydrazide) **1**, nicotinaldehyde, and tetranicotinaldehyde palladium(II) nitrate involving Pd–pyridine and hydrazone bond formation simultaneously. In all cases, the synthesis of cage **C1**·(NO_3_)_4_ was performed in deuterated DMSO at 25 °C, and cage formation was monitored by ^1^H NMR spectroscopy. The solvent DMSO was chosen as it enables the complete solution of the reagents, cage, and reaction intermediates. Attempts to use fewer coordinating solvents (e.g., methanol or chloroform) resulted in low solubility of the reagents, and also, in the formation of precipitates that hamper both cage formation and the quantification of the species in solution. Quantification of the concentration of the species in solution was performed by integration of the corresponding proton signals considering the concentration of the internal standard 1,4-dimethoxybenzene. The same procedure was used consistently for determining cage formation yield.

**Figure 3:**
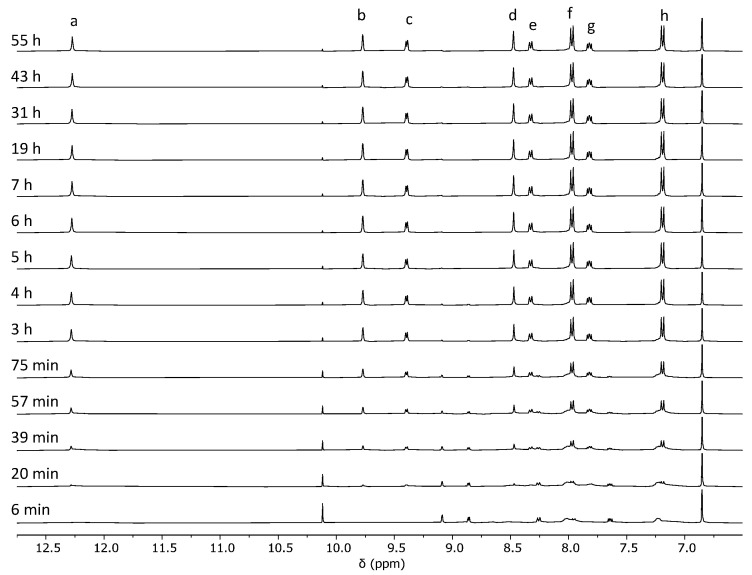
Evolution of the ^1^H NMR (400 MHz, DMSO-*d*_6_) for the synthesis of cage **C1**·(NO_3_)_4_ through reaction pathway 1 from **1** and **2**·(NO_3_)_2_. The signal at 6.86 ppm corresponds to 1,4-dimethoxybenzene used as an internal standard. The assignment of cage signals a–h is shown in Figure 2. Due to the complexity of the cage formation reaction, the signals of building blocks and intermediates are not assigned. We were only able to assign the set of signals at 10.1, 9.1, 8.9, 8.3, and 7.6 ppm to nicotinaldehyde.

Initially, we performed the synthesis of cage **C1** through reaction pathway 1. For this, we placed dihydrazide **1** and tetranicotinaldehyde palladium(II) nitrate **2**·(NO_3_)_2_ in an NMR tube and monitored the evolution of the reaction by acquiring ^1^H NMR spectra at different time intervals for a total of 55 h (Figure 3 and Figure S1). The signals corresponding to the starting materials disappeared rapidly with the simultaneous formation of the signals of the Pd_2_L_4_ cage. A set of signals corresponding to free nicotinaldehyde forms quickly at the beginning of the reaction and then disappears over time, suggesting a complex cage formation mechanism. In contrast, few signals of the reaction intermediates could be observed in the reaction mixture, probably due to a combination of the formation of a large number of reaction intermediates in fast exchange with unsymmetrical structures containing chemically inequivalent NMR signals, as often observed in cage formation reactions [43,77].

**Figure 4:**
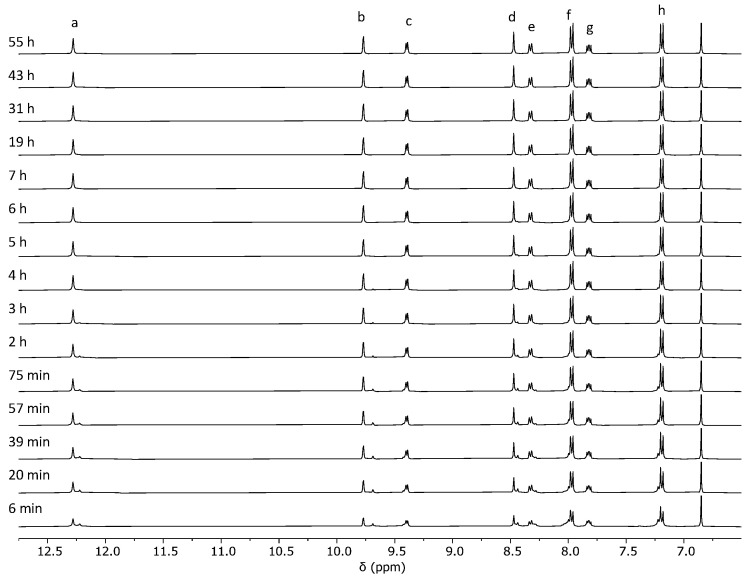
Evolution of the ^1^H NMR (400 MHz, DMSO-*d*_6_) for the synthesis of cage **C1**·(NO_3_)_4_ through reaction pathway 2 from **3** and palladium(II) nitrate dihydrate. The signal at 6.86 ppm corresponds to 1,4-dimethoxybenzene used as an internal standard. The assignment of cage signals a–h is shown in Figure 2. Due to the complexity of the cage formation reaction, the signals of building blocks and intermediates are not assigned.

Then, we performed the synthesis of cage **C1** through reaction pathway 2. We reacted dihydrazide ligand **3** with palladium(II) nitrate dihydrate to form the cage through Pd–pyridine bond formation. The reaction proceeds smoothly with the formation of cage **C1**, which is the only product observed in the ^1^H NMR spectra (Figure 4 and Figure S2). In contrast to reaction pathway 1, after 6 min, nearly all signals corresponding to the starting materials are absent, and the predominant signals in the spectra correspond to the cage. This highlights the rapid formation of the Pd–pyridine bonds, resulting in quick cage formation.

**Figure 5:**
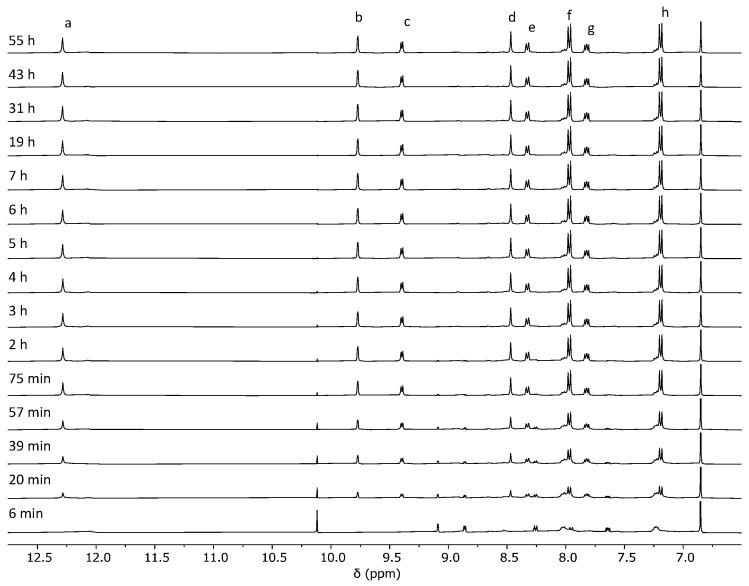
Evolution of the ^1^H NMR (400 MHz, DMSO-*d*_6_) for the synthesis of cage **C1**·(NO_3_)_4_ through reaction pathway 3 from dihydrazide **1**, palladium(II) nitrate dihydrate, and nicotinaldehyde. The signal at 6.86 ppm corresponds to 1,4-dimethoxybenzene used as an internal standard. The assignment of cage signals a–h is shown in Figure 2. Due to the complexity of the cage formation reaction, the signals of building blocks and intermediates are not assigned. We were only able to assign the set of signals at 10.1, 9.1, 8.9, 8.3, and 7.6 ppm to nicotinaldehyde.

Finally, we performed the synthesis of cage **C1** through reaction pathway 3, which involves dihydrazide **1**, palladium(II) nitrate dihydrate, and nicotinaldehyde. This reaction is more complex, as it involves the simultaneous formation of 16 bonds, comprising 8 Pd–pyridine bonds and 8 hydrazone bonds. Indeed, this reaction pathway involves more difficulty; for example, hydrazine and hydrazone groups may result in competition with pyridine for Pd^2+^ coordination that may disturb the cage formation pathway [78,79,80,81]. This is supported by the free nicotinaldehyde signals observed at the beginning of the reaction, which disappear over time, suggesting that slow ligand displacement reactions are occurring. However, despite this complexity, the reaction yields the expected **C1** cage in a clean formation reaction (Figure 5 and Figure S3). This experiment highlights the feasibility of the simultaneous formation of Pd–pyridine and hydrazone bonds in the cage formation reaction.

### 2.2. Analysis of the Cage Formation Reactions

**Figure 6:**
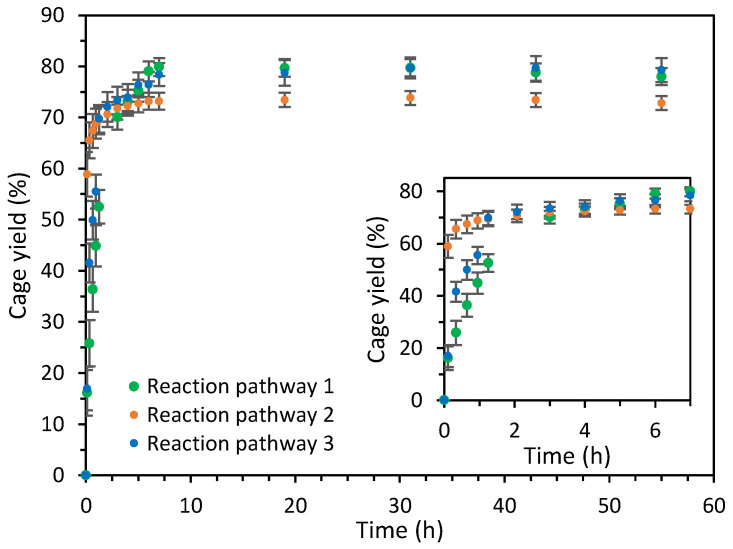
Evolution of the cage **C1**·(NO_3_)_4_ yield for the cage formation reaction through reaction pathways 1 (green), 2 (orange), and 3 (blue). The inset plot shows the first 7 h of reaction. Cage formation yields have been determined by ^1^H NMR using the integrals of the signals of the cage and 1,4-dimethoxybenzene which has been used as an internal standard.

After performing the cage formation reactions, we carried out a quantitative analysis of the integrals of the ^1^H NMR signals for the three reaction pathways to evaluate the kinetics of cage formation. For this, the integral of the internal standard 1,4-dimethoxybenzene was taken into account (Figure 6 and Table S1). We observed that the fastest cage formation is for reaction pathway 2, which gives a 65% yield in 6 min. In contrast, reaction pathways 1 and 3, only give a 16% and 17% yield in 6 min, respectively. Considering that reaction pathway 1 involves the reaction of 6 building blocks (4 molecules of dihydrazide **1** and 2 molecules of tetranicotinaldehyde palladium(II) nitrate **2**·(NO_3_)_2_) through hydrazone bond formation, and reaction pathway 2 involves the reaction of 4 building blocks **3** and 2 Pd(II) atoms through Pd–pyridine bond formation. These results highlight that the formation of the Pd–pyridine bond has a greater rate than the formation of the hydrazone bond. A key observation, which involves the formation of 16 bonds through the reaction of 12 building blocks and 2 Pd(II) atoms, is that it exhibits similar reaction kinetics to reaction pathway 1, which only requires the formation of 8 bonds from the reaction of 6 building blocks. This observation shows that hydrazone bond formation is the rate-limiting step of the cage formation process, in contrast to the fast Pd–pyridine bond formation. Focusing on the final cage formation yield at 55 h of reaction, both reaction pathways 1 and 3 have similar yields in the range of 78–79%, whereas reaction pathway 2 has a 73% yield. Despite all that, reaction pathway 2 is the fastest, and the final yield is the lowest, highlighting that the final yield does not depend exclusively on the initial reaction rate. As the cage formation reaction yields are less than 100% for the three reaction pathways, in all three cases, by-products are formed. 

**Figure 7:**
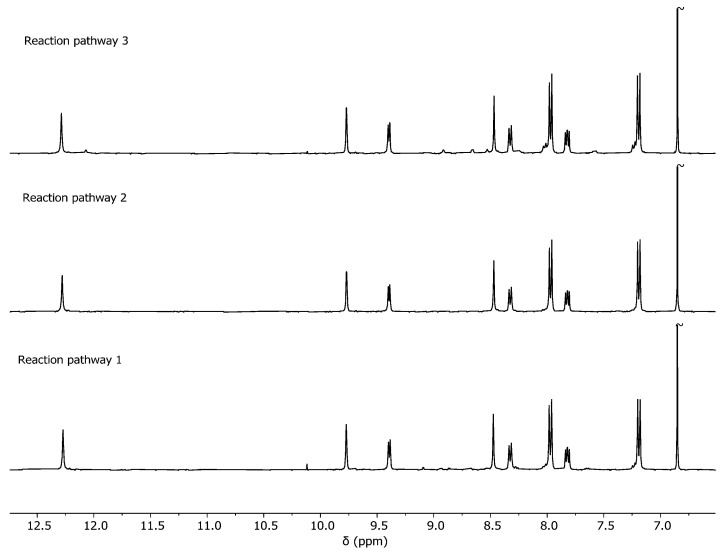
Comparison of the ^1^H NMR obtained at 55 h for the formation reaction of cage **C1**·(NO_3_)_4_ through reaction pathways 1, 2, and 3. The signal at 6.86 ppm corresponds to 1,4-dimethoxybenzene used as an internal standard. The signal at 10.1 ppm corresponds to the aldehyde group of unreacted nicotinaldehyde.

In order to determine if the by-products formed in the three reaction pathways are visible by ^1^H NMR, a close examination of the obtained spectra at the end of the reaction was performed. While reaction pathways 1 and 2 produce clean ^1^H NMR spectra showing only the signals of cage **C1**, reaction pathway 3 displays a small set of additional peaks (Figure 7). This is probably due to the formation of asymmetric oligomeric structures possessing a number of chemically distinct NMR signals that are individually at too low concentration to be observed by ^1^H NMR.

### 2.3. Molecular Modeling

**Figure 8:**
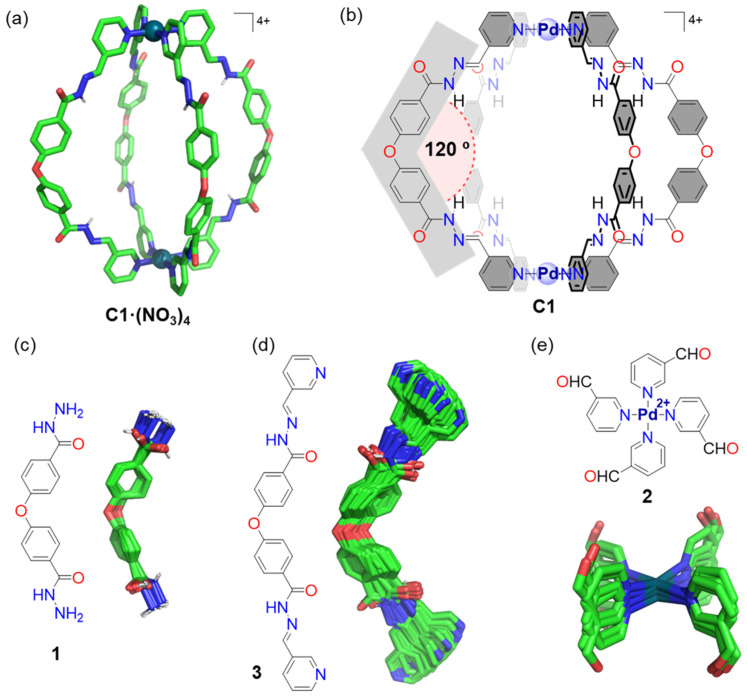
(**a**) Crystal structure of cage **C1**·(NO_3_)_4_ (CCDC 2295536, see ref [31]). (**b**) Chemical representation of the structure of cage **C1** highlighting the ideal 120° angle of the ligand. (**c**–**e**) Conformational searches performed at MMFF level of theory using the software Wavefunction Spartan 20 (overlay of the most stable conformers found in a 2 kcal/mol energy window). The molecular models, in which non-polar hydrogen atoms have been omitted for clarity, have the following color scheme: C, green; O, red; N, blue; H, white; and Pd^2+^, dark cyan.

To understand the successful cage formation observed in the three different reaction pathways, we also performed molecular mechanics calculations. We carried out a conformational search for each of the cages’ building blocks to identify the most stable conformations. We observed that all conformations of ligands **1** and **3** have a bend configuration (Figure 8c,d), with good complementarity of ligand **1** to the geometry of metal complex **3** (Figure 8e) and ligand **3** to the square planar geometry of Pd(II). Specifically, the rigidity of the core Ph–O–Ph fragment of ligands **1** and **3** provides a key structural element with an average bend angle of 121° (Figure 8c) and 118° (Figure 8d), respectively. These angles match nicely the 130 °C average angle the ligand has in the crystal structure of cage **C1**·(NO_3_)_4_ (Figure 8a) and the theoretical bend angle of 120° of the chemical representation of cage **C1** (Figure 8b). This analysis suggests that the successful cage formation from the ligands is linked to a favorable preorganization of the building blocks, whose geometry aligns well with the cage structure.

## 3. Materials and Methods

Materials. All chemicals and solvents were obtained from commercial sources and used without further purification unless specified.

NMR Experiments. ^1^H spectra were recorded on a Bruker FT-NMR Avance 400 (Ettlingen, Germany) spectrometer at 300 K. Chemical shifts (δ) are reported in parts per million (ppm) and referenced to residual solvent peak.

Molecular Modeling. The structure of ligands was modeled with the Spartan’ 20 software, using the built-in conformational search algorithm using the MMFF force field [82].

### 3.1. Synthesis of Ligands and Cages

Compounds **1**, **2**·(NO_3_)_2_, **3**, and **C1**·(NO_3_)_4_ were prepared as described by our research group as reported in the literature [31].

### 3.2. Cage Formation Kinetic Experiments

All ^1^H NMR kinetic experiments were performed using the following general procedure. To an NMR tube, **1** (2.6 mg, 9.2 µmol) and **2**·(NO_3_)_2_ (3.0 mg, 4.6 µmol) were introduced for reaction pathway 1; **3** (4.3 mg, 9.2 µmol) and Pd(NO_3_)_2_·2H_2_O (1.2 mg, 4.6 µmol) for reaction pathway 2; or **1** (2.6 mg, 9.2 µmol), and Pd(NO_3_)_2_·2H_2_O (1.2 mg, 4.6 µmol) for reaction pathway 3. Then, a stock solution of 1,4-dimethoxybenzene as an internal standard in DMSO-*d*_6_ (600 µL of a 10 mM stock solution) was added for reaction pathways 1 and 2. For reaction pathway 3, a solution of 1,4-dimethoxybenzene as internal standard (600 µL of a 10 mM stock solution) containing nicotinaldehyde (1.8 µL, 18.4 mmol). The reaction was shaken to obtain a clear solution of all the components, and the crude reaction mixture was monitored by ^1^H NMR for 55 h at 25 °C. The concentration of all chemical species was determined for each reaction time by the analysis of integrals of the ^1^H NMR signals of the cage and the internal standard, reporting the yield as the average. All reactions were performed at least twice, and a representative example is reported in the manuscript.

## 4. Conclusions

We have performed a study of the synthesis of a Pd_2_L_4_ hydrazone molecular cage **C1** (**C1** = [Pd_2_L_4_]^4+^ with nitrate counterions, i.e., **C1**·(NO_3_)_4_) through 3 different reaction pathways involving the formation of Pd–ligand bonds, hydrazone bonds, or a combination of both. Our results show that it is possible to synthesize the cage structure **C1** through three different reaction pathways, obtaining yields ranging from 73 to 79%, with the lowest yield observed for pathway 2 (73%) and similar yields for pathways 1 and 3 (78% and 79%, respectively). The fastest initial reaction rate is observed for reaction pathway 2, compared to reaction pathways 1 and 3, which have similar initial reaction rates, indicating that Pd-pyridine bonds are formed faster than hydrazone bonds. Overall, the cage formation pathway influences the initial reaction kinetics and the final cage yield. We also proved that despite the complexity of reaction pathway 3, which involves the formation of 16 bonds in contrast to reaction pathways 1 and 2, which only involve the formation of 8 bonds, the cage is formed in a 79% yield. Molecular modeling shows that the ligands have a favorable preorganization and their geometry matches well with the cage structure. We anticipate that these results will open the way for more complex cage designs that involve reaction pathways with the simultaneous formation of both Pd–ligand and hydrazone bonds.

## Data Availability

The original contributions presented in the study are included in the article/Supplementary Materials, further inquiries can be directed to the corresponding author/s.

## Supplementary Materials

The following supporting information can be downloaded at: https://www.mdpi.com/article/10.3390/ijms252211861/s1.
