# Supplementary material for: Synthesis of a Pd2L4 Hydrazone Molecular Cage Through Multiple Reaction Pathways
Source: Int J Mol Sci. 2024 Nov 5;25(22):11861. doi: 10.3390/ijms252211861 (PMC11593401; doi:10.3390/ijms252211861)
Supplement: Supplementary file 1 [file ijms-25-11861-s001.zip › ijms-3285018-supplementary.pdf]

Supporting Information

Synthesis of a Pd_2L_4 hydrazone molecular cage through multiple reaction pathways

Giovanni Montà-González, Ramón Martínez-Máñez,* and Vicente Martí-Centelles *

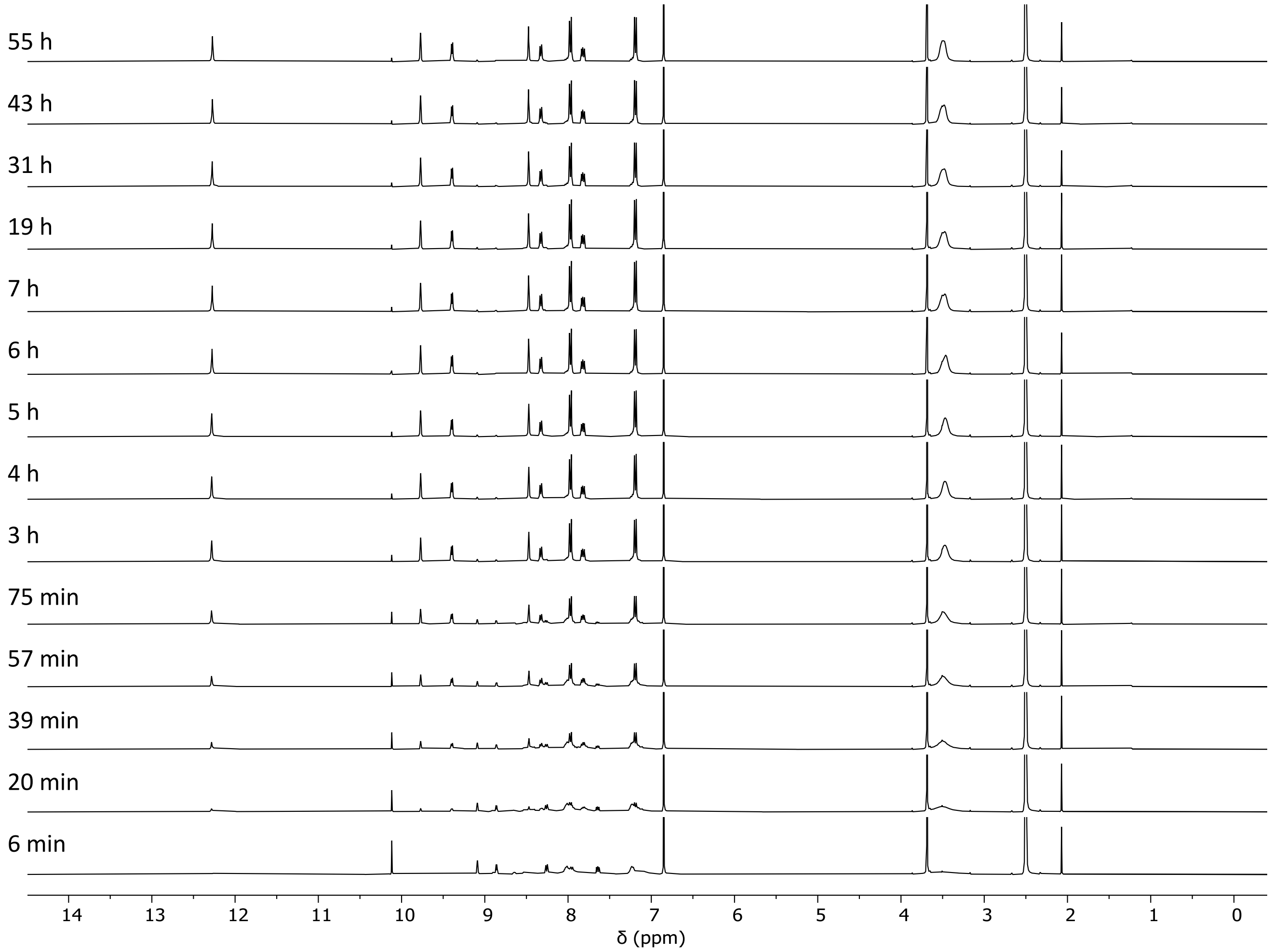

Figure S1. Evolution of the ^{1}H NMR (400 MHz, DMSO-d_6) for the synthesis of cage **C1**·$(NO_3)_4$ through reaction pathway 1 from **1** and **2**·$(NO_3)_2$. The signals at 3.69 ppm and 6.86 ppm correspond to 1,4-dimethoxybenzene used as an internal standard.

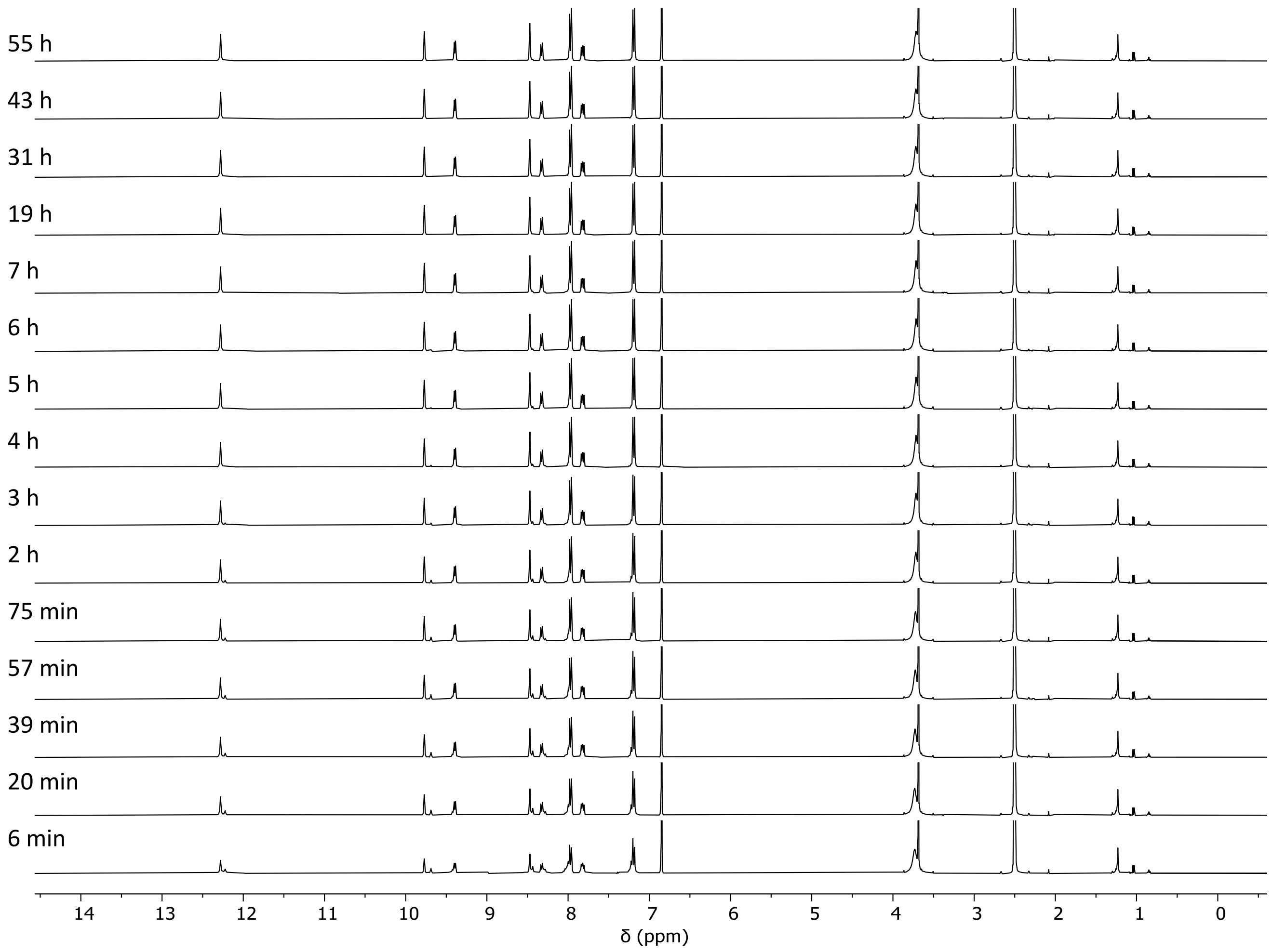

Figure S2. Evolution of the ^{1}H NMR (400 MHz, DMSO-d_6) for the synthesis of cage **C1**·$(NO_3)_4$ through reaction pathway 2 from **3** and palladium(**II**) nitrate dihydrate. The signals at 3.69 ppm and 6.86 ppm correspond to 1,4-dimethoxybenzene used as an internal standard.

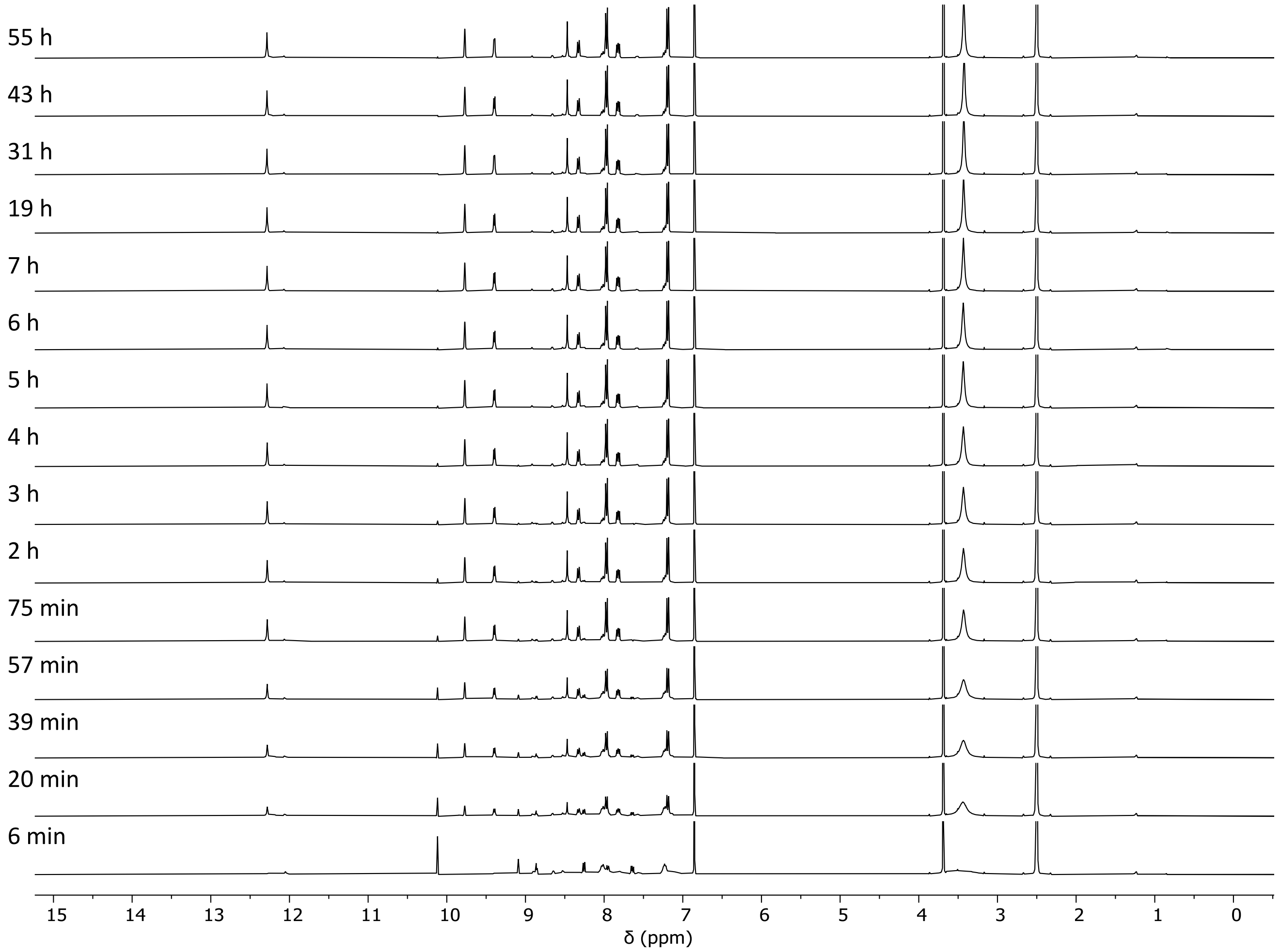

Figure S3. Evolution of the ^{1}H NMR (400 MHz, DMSO-*d*$_6$) for the synthesis of cage **C1**·$(NO_3)_4$ through reaction pathway 3 from dihydrazide **1**, palladium(II) nitrate dihydrate, and nicotinaldehyde. The signals at 3.69 ppm and 6.86 ppm correspond to 1,4-dimethoxybenzene used as an internal standard.

Table S1. Cage **C1**·$(NO_3)_4$ formation yields determined by ^{1}H NMR using 1,4-dimethoxybenzene as internal standard. [a] Not determined due to a problem in the NMR acquisition.

Time	Reaction pathway 1		Reaction pathway 2		Reaction pathway 3	
	Yield (%)	Error (%)	Yield (%)	Error (%)	Yield (%)	Error (%)
6 min	16.2	4.4	58.9	4.4	17.0	4.2
20 min	25.8	4.6	65.5	3.5	41.6	3.8
39 min	36.4	4.4	67.3	3.3	49.9	3.8
57 min	44.9	4.0	68.8	2.9	55.5	3.3
75 min	52.5	3.3	69.4	2.7	69.7	2.7
124 min	– [a]	– [a]	70.6	2.4	72.1	2.9
3 h	70.1	2.5	71.8	2.2	73.3	2.7
4 h	73.1	2.3	72.3	1.9	73.9	2.6
5 h	75.3	2.2	72.7	1.7	76.4	2.4
6 h	79.1	1.9	73.2	1.6	76.4	2.5
7 h	79.9	1.7	73.2	1.6	78.4	2.2
19 h	79.7	1.7	73.4	1.4	78.7	2.5
31 h	79.7	1.7	73.8	1.4	79.7	2.0
43 h	78.8	1.8	73.4	1.4	79.6	2.4
55 h	78.0	1.6	72.8	1.4	79.3	2.4
